# Atrial fibrillation incidence and impact of biventricular pacing on long-term outcome in patients with heart failure treated with cardiac resynchronization therapy

**DOI:** 10.1186/s12872-019-1169-1

**Published:** 2019-08-13

**Authors:** Jonatan Jacobsson, Christian Reitan, Jonas Carlson, Rasmus Borgquist, Pyotr G. Platonov

**Affiliations:** 1 Department of Cardiology, Clinical Sciences, Lund University, 221 85 Lund, Sweden; 2 Department of Medicine, Sahlgrenska University Hospital, Mölndal, Sweden; 3 Arrhythmia Clinic, Skåne University Hospital, Lund, Sweden; 4 Department of Cardiology, Aarhus University Hospital, Aarhus, Denmark

**Keywords:** Cardiac resynchronization therapy, Biventricular pacing, Atrial fibrillation, Atrial high rate episodes, Mortality, Heart failure

## Abstract

**Background:**

In patients with cardiac resynchronization therapy (CRT), atrial fibrillation (AF) is associated with an unfavorable outcome and may cause loss of biventricular pacing (BivP). An effective delivery of BivP of more than 98% of all ventricular beats has been shown to be a major determinant of CRT-success.

**Methods:**

At a Swedish tertiary referral center, data was retrospectively obtained from patient registers, medical records and preoperative electrocardiograms. Data regarding AF and BivP during the first year of follow-up was assessed from CRT-device interrogations. No intra-cardiac electrograms were studied. Kaplan-Meier curves and Cox-regression analyses adjusted for age, etiology of heart failure, left ventricular ejection fraction, left bundle branch block and NYHA class were performed to assess the impact of AF and BivP on the risk of death or heart transplantation (HTx) at 10-years of follow-up.

**Results:**

Preoperative AF-history was found in 54% of the 379 included patients and was associated with, but did not independently predict death or HTx. The one-year incidence of new device-detected AF was 22% but not associated with poorer prognosis. At one-year, AF-history and BivP≤98%, was associated with a higher risk of death or HTx compared to patients without AF (HR 1.9, 95%CI 1.2–3.0, *p* = 0.005) whereas AF and BivP> 98% was not (HR 1.4, 95%CI 0.9–2.3, *p* = 0.14).

**Conclusions:**

In CRT-recipients, AF-history is common and associated with poor outcome. AF-history does not independently predict mortality and is probably only a marker of a more severe underlying disease. BivP≤98% during first-year of CRT-treatment independently predicts poor outcome thus further supporting the use of 98% threshold of BivP, which should be attained to maximize the benefits of CRT.

## Background

There is a strong evidence that cardiac resynchronization therapy (CRT) reduces mortality and morbidity in patients with chronic heart failure (HF), depressed left ventricular ejection fraction (LVEF), widened QRS-complex and signs of electrical dyssynchrony in patients with sinus rhythm (SR) [1, 2].

A history of preoperative atrial fibrillation (AF) has been found to be associated with unfavorable outcome and higher risks of non-response to treatment [3, 4]. However, the effect of CRT in patients with AF is still not fully understood and in the current guidelines, AF has been identified as a gap in knowledge with need for further studies [5]. AF is the most common arrhythmia in patients with HF, and its prevalence is directly linked to the severity of HF and up to 50% of patients with an advanced disease have AF [4, 6]. In a European Clinical practice registry, 23% of patients who received CRT were in AF [4].

An effective delivery of biventricular pacing (BivP) has been shown to be a major determinant of the success of CRT [4] and AF may cause loss of BivP [7]. Previous studies have defined high BivP as > 98% of all ventricular beats and in large-scale studies, the greatest magnitude of reduction in mortality has been observed in patients with a BivP achieved in excess of 98% [8, 9].

Designed to prevent tracking of rapidly occurring signals sensed by atrial channels to the ventricles, automatic mode switching (AMS) algorithms have been used for the detection of atrial tachyarrhythmias, often referred to as atrial high-rate episodes (AHREs), with high sensitivity and specificity for AF [10]. As CRT-devices are capable of detecting and storing AHREs and AMS-events during follow-up, the prognostic impact of these parameters can be studied but there are conflicting results regarding the impact of such episodes [11, 12].

To estimate the prevalence and incidence of AF in epidemiology studies, data from national discharge registers are commonly used. Even though specificity of AF diagnosis in the Swedish National Patient Register (SNPR) is reported to be high [13], the AF diagnosis in the SNPR has recently been found to underestimate the prevalence of AF by at least 20% [14].

The objectives of this study were to evaluate the prognostic impact of pre-procedural AF history in patients with CRT and to assess the one-year incidence and prognostic importance of new onset device-detected AF. In patients with documented AF by one-year after CRT-implantation we also aimed to evaluate the impact of achieved BivP during the first year of CRT-treatment on the risk of death or heart transplantation at 10 years of follow-up. We hypothesized that BivP≤98% would be associated with poorer outcome.

## Methods

### Study population

Consecutive patients who received CRT device with (CRT-D) or without (CRT-P) defibrillator function during the period of 1999–2008 at a tertiary care center (Skane University Hospital) were identified through a registry. Demographical, clinical characteristics and data from the follow-up CRT device-interrogations were retrieved from medical records. Patients fulfilling contemporary European Society of Cardiology’s guideline indications for CRT [4] at the time of CRT implantation with a successfully implanted device were included. The local ethics committee approved the study.

### Study endpoint

Endpoint was defined as death from any cause censored for heart transplantation or combined endpoint of death from any cause or heart transplantation and assessed by record linkage with the Swedish Cause of Death Registry and the SNPR. Patients were followed 10 years from the time of CRT implantation, until the endpoint, or until 25th of May 2013 when data was retrieved from the SNPR.

### Assessment of pre-procedural atrial fibrillation

Information concerning AF-history was acquired by record linkage with the SNPR, by reviewing medical records and the regional digital ECG archive. The SNPR is administered by the Swedish National Board of Health and Welfare and includes data, starting in the year 1987 on main and secondary diagnoses at discharge from all public hospitals in Sweden. Information about outpatient visits to hospitals is also included. The register uses International Classification of Disease (ICD) codes, with the 9th edition (ICD-9) used between 1987 and 1996, and the 10th edition (ICD-10) used from 1997. AF was defined as the presence of any of the following ICD codes: 427D for ICD-9 and I48 for ICD-10.

To study if there were any additional AF-cases not reflected in the SNPR or medical records, all available preoperative electrocardiograms (ECGs) of the included patients without AF-history prior to CRT implantation were manually reviewed by a trained physician (J.J.) and validated by a senior cardiologist (P.P.) in case of doubts. On surface ECG, AF was defined as a rhythm disorder with irregular RR intervals, indistinct P-waves, and atrial cycle length of 200 ms where distinct atrial activity was visible on surface ECG [15]. AF documentation was based on ECG data obtained from the regional electronic ECG databases (MUSE Cardiology Information System v9, GE Healthcare, Chicago, Illinois and Infinity Megacare ECG management system, Dräger, Houston, Texas), which contain all ECGs taken in the hospital catchment area, including primary care facilities, starting from the year 1988.

Patients were considered to have AF history if it was documented by the SNPR or/and by medical records or/and on any available ECG at any time before CRT implantation. Depending on the presence and clinical type of AF, all patients were classified as having either history of non-permanent AF, permanent AF or no AF history.

### Assessment of post-operative device-detected data

Data from routine CRT device interrogations after CRT implantation were evaluated to assess AF-incidence and achieved BivP in percent of all ventricular beats during first year of follow-up. Please note that two independent analyses were made in this study. One was based on the impact of pre-procedural AF and based exclusively on the information available by the time of device implantation thus including all patients enrolled in this registry study. The second analysis was aimed at assessment of the extent of achieved biventricular pacing and information regarding device-detected AF and therefore could only be performed on a subset of the CRT population as described below.

Only patients who had available device-detected data after 3 months from CRT implantation and who did not reach the combined endpoint during the first year of follow-up were included in the analyses regarding device-detected AF and BivP. AMS episodes and AHREs were considered as episodes of device-detected AF and will be described as this henceforth in this article. No analyses regarding the number of, or total duration of the episodes were made. If device-detected AF was found in a patient during first year of follow-up, this patient was considered to have documented non-permanent AF-history by one year after CRT implantation. Manufacturer-specific nominal settings for the detection of AMS and AHREs were used as default.

Regarding the assessment of achieved BivP, patients were only included if achieved BivP was documented in over 50% of the patients’ available CRT-device interrogations during the first year of follow-up.

### Statistics

The Kolmogorov–Smirnov test and histograms were used to evaluate if continuous data were normally distributed or not. Non-continuous and continuous variables not normally distributed were reported as median ± quartiles. Continuous, normally distributed variables were reported as mean ± standard deviation (SD). The t-test and Mann Whitney U-test was used to analyze differences among normally and not normally distributed samples respectively. The Kruskal–Wallis test was used to evaluate if distributions were the same across groups and the Pearson chi-square test was used to compare categorical variables.

A uni- and a multivariate Cox-regression analysis was used to assess the impact of AF-history before CRT implantation, new onset device-detected AF and the extent of BivP dichotomized by 98% during first year of follow-up on the combined endpoint as well as total mortality censored for heart transplant. The Cox model was adjusted for clinical covariates known to be associated with the endpoint and with *p*-values ≤0.150 in the univariate Cox-regression analysis; age, ischemic etiology of HF, LVEF, new york heart association (NYHA) Class III/IV and left bundle branch block (LBBB) [2].

Kaplan–Meier plots and log-rank tests were used to compare survival over time between groups. SPSS Statistics for Macintosh, version 24.0 (IBM Corp., Armonk, NY) was used for all statistical analyses.

## Results

The study included 379 consecutive patients with CRT-P or CRT-D treatment (median age 71 years, 85% males).

Data obtainment from the SNPR and medical records revealed a documented AF history before CRT-implantation in 189 of the 379 included patients (50%). Manual assessment of all available ECGs prior to CRT implantation among all patients without AF history according to the SNPR or medical records (*n* = 190) revealed further 17 AF-cases and after combining the three sources (the SNPR, medical records and all available preoperative ECGs) 206 patients (54%) had preoperative AF history. Consequently, 8% of all patients with AF history before CRT implantation had AF history based only on ECG prior to CRT implantation, but not officially recorded in the SNPR or medical records. A total of 4975 preoperative ECGs were reviewed, with a median number of 22 (IQR 11–31) ECGs per patient.

Baseline clinical data is presented in Table 1. Patients with AF history were older and achieved less satisfactory BivP compared to patients with no AF. Among patients with AF and low BivP the median BivP was 93% (IQR 82–96) during first year of follow-up. Patients with AF had a higher likelihood of being treated with digoxin, warfarin or class I or III antiarrhythmic drugs and they were more often treated with a conventional pacemaker before CRT treatment. LBBB was more common among patients with no AF history. No differences between the groups could be observed regarding customary HF medications or significant co-morbidities such as diabetes and hypertension or preoperative plasma creatinine concentration. In the non-permanent AF group the median AF burden first year of CRT treatment was 0% (IQR 0–15). Twenty-one patients underwent AV-junctional ablation before and nine after CRT implantation (18 with permanent, 11 with non-permanent and 1 with new-onset device-detected AF).

**Table 1:** Characteristics of all included patients

Characteristics of Patients	All (*n* = 379)	No AF (*n* = 173, 46%)	AF (*n* = 206, 54%)	No AF vs. AF
Demographics at CRT implantation				
Age (IQR)	71 (62–76)	69 (59–75)	72 (66–76)	**0.026**
Male	85%	81%	88%	**0.04**
Ischemic heart disease	57%	55%	59%	0.40
NYHA Class III or IV	91%	89%	92%	0.34
Hypertension	36%	36%	35%	0.8
Diabetes	34%	32%	35%	0.5
Conventional PM before CRT	24%	17%	30%	**0,003**
Creatinine (IQR)	110 (90–137)	110 (90–136)	114 (89–139)	0.57
Medication at CRT implantation				
β-Blocker	82%	82%	82%	0.9
ACEi or ARB	94%	96%	93%	0.16
Loop diuretic	92%	92%	93%	0.7
Class I or III antiarrhythmic	14%	8%	29%	**0.003**
Digoxin	37%	30%	42%	**0.01**
Warfarin	55%	32%	75%	**< 0.001**
Cardiac findings at CRT implantation				
QRS Duration ms (IQR)	170 (154–184)	170 (154–184)	170 (152–184)	0.98
LV ejection fraction (IQR)	22 (20–25)	22 (20–25)	22 (20–27)	0.98
LBBB	62% (236)	72% (125)	54% (53)	**< 0.001**
Follow-up characteristics				
CRT-P implanted	74%	75%	73%	0.78
Median follow-up months (IQR)	40 (11–83)	59 (23–89)	30 (5–76)	**< 0.001**
BivP first year of follow-up 98% or less	35%	22%	48%	**< 0.001**
Death during follow-up	232 (61%)	88 (51%)	144 (70%)	**< 0.001**
HTx during follow-up	13 (3%)	6 (3%)	7 (3%)	0.97

Significant *p*-values, < 0,05, in bold

The number of deaths during follow-up was 232 and 13 patients were heart transplanted. In total, 245 patients (65%) reached the endpoint during follow-up, 73% of patients with AF-history and 54% of patients with no AF-history (log rank *p* < 0.001).

### Prognostic impact of pre-procedural atrial fibrillation

**Fig. 1:**
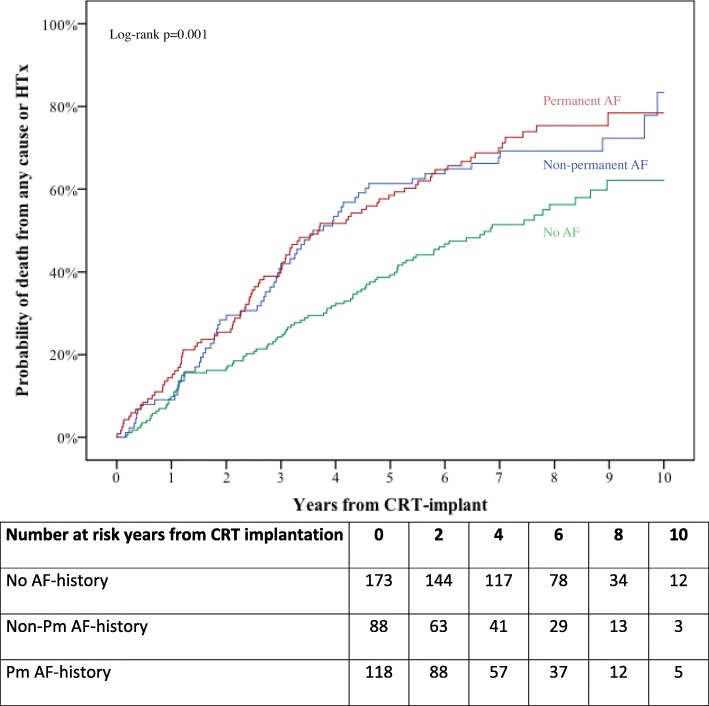
Kaplan-Meier analysis, stratified for history of no AF, non-permanent AF or permanent AF before CRT-implant. Follow-up time is 10 years from CRT implantation. Abbreviations: AF = atrial fibrillation, CRT = cardiac resynchronization therapy, HTx = heart transplantation

AF history before CRT implantation was associated with unfavorable outcome but no significant differences in outcome were found when patients with non-permanent AF and permanent AF were compared (Fig. 1). In the univariate Cox regression analysis pre-procedural AF was significantly associated with the combined endpoint in the univariate but not in the multivariate model (Table 2). No significant association between the pre-procedural AF history and death from any cause at 10 years of follow-up was observed in a multivariate model adjusted for age, ischemic etiology, LVEF, LBBB and NYHA Class (HR 1.26, 95%CI 0.93–1.70, *p*-value = 0.133).

**Table 2:** Uni- and multivariate Cox regression anaysis. Follow-up time is 10 years from CRT implantation

Variable	Univariate cox regression analysis, risk of death from any cause or heart transplantation			Multivariate cox regression analysis, risk of death from any cause or heart transplantation		
	HR	95%CI	*p*-value	Adjusted* HR	95%CI	*p*-value
At CRT implantation						
Age	1.03	1.02–1.05	**< 0.001**	1.02	1.001–1.03	**0.03**
Pre-procedural AF vs. No AF (ref)	1.66	1.28–2.16	**< 0.001**	1.34	0.99–1.80	0.055
Male vs. Female (ref) gender	0.92	0.64–1.32	0.63			
Ischaemic etiology of HF	1.70	1.30.2.21	**< 0.001**	1.49	1.10–2.02	**0.009**
NYHA Class III/IV vs. I/II (ref)	1.90	1.08–3.33	**0.03**	1.87	1.07–3.29	**0.03**
QRS Duration (ms)	1.00	1.00–1.01	0.68			
LV ejection fraction (%)	0.97	0.95–0.99	**0.006**	0.98	0.96–0.998	**0.003**
LBBB vs. Non-LBBB (ref)	0.74	0.58–0.96	**0.02**	0.90	0.67–1.20	0.453
CRT-D vs. CRT-P (ref)	0.83	0.62–1.11	0.21			

* adjusted for age, pre-procedural AF, NYHA Class I/II compared to III/IV, LVEF, LBBB and ischemic etiology of HF at CRT implantation

Significant *p*-values, < 0,05, in bold

### Incidence and prognostic impact of device-detected atrial fibrillation during the first year of follow-up

In total, 144 (83%) of all patients with no AF preoperatively had sufficient follow-up data (see methods section) to be included this analysis. Of them, 31 (22%) had device-detected AF during first year of follow-up, so that the total prevalence of either pre-procedural or device-detected AF adds up to 63% of all patients by one year after CRT implantation.

Among patients without AF history prior to CRT implantation, device-detected new-onset AF during first year of follow-up was not significantly associated with the outcome (Table 3).

**Table 3:** Adjusted multivariate Cox regression analysis. Follow-up time is one year from CRT implantation to 10 years from CRT implantation

Variable	Risk of death from any cause or heart transplantation		
	Adjusted HR*	95%CI	p-value
One year after CRT implantation			
New-onset AF vs. No AF (ref)	1.65	0.89–3.09	0.12
AF + BivP> 98% vs. No AF (ref)	1.42	0.89–2.26	0.14
AF + BivP≤98% vs. No AF (ref)	1.93	1.23–3.03	**0.005**

* adjusted for age, pre-procedural AF, NYHA Class I/II compared to III/IV, LVEF, LBBB and ischemic etiology of HF at CRT implantation

Significant *p*-values, < 0,05, in bold

#### Prognostic impact of achieved biventricular pacing during the first year of follow-up

Of all patients, 254 (67%) had sufficient follow-up information to be included in the analyses regarding the association of BivP with prognosis. Of those, 35% had BivP≤98% during the first year of follow-up. Patients with AF had significantly less BivP (Table 1).

There were no significant differences regarding the demographics, medications or clinical characteristics at CRT implantation when patients with AF and BivP≤98% and patients with AF and BivP> 98% were compared. Also, no difference in the heart rate at baseline could be found (*p* = 0.57).

**Fig. 2:**
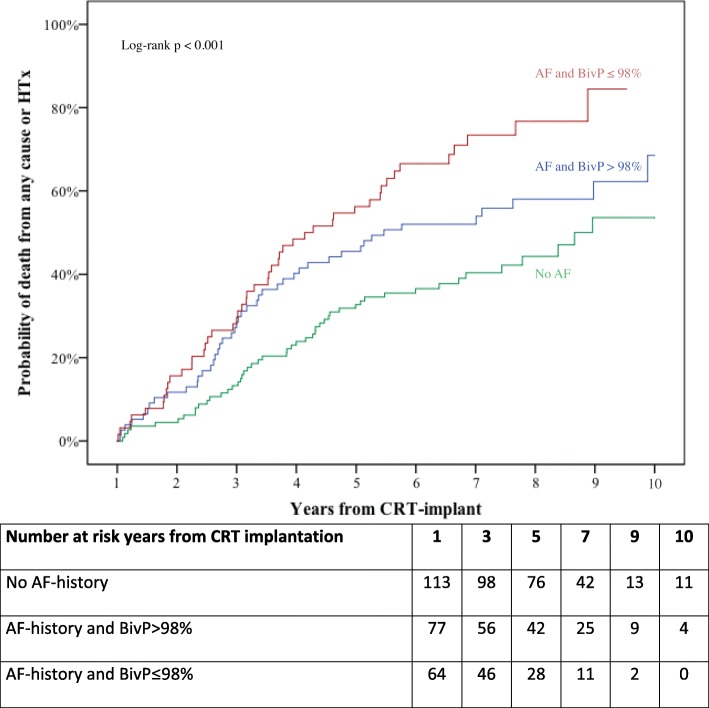
Kaplan Meier stratified for groups No AF-history one year after CRT implantation, AF-history one year after CRT implantation and BivP> 98% during first year of follow-up and AF-history one year after CRT implantation and BivP≤98% during first year of follow-up. Follow-up time is one year from CRT implantation to 10 years from CRT implantation. Abbreviations: AF = atrial fibrillation, BivP = biventricular pacing, CRT = cardiac resynchronization therapy, HTx = heart transplantation

At one-year of follow-up, BivP≤98% in patients with either permanent or non-permanent AF was associated with a significantly worse outcome compared to patients with no AF (Fig. 2) and was independently associated with the outcome, whereas AF patients with BivP> 98% had similar prognosis compared to patients with no AF (Table 3).

When only patients with implanted CRT-P device were analyzed in the multivariate analysis adjusted for the same variables as in Table 3, BivP≤98% remained an independent predictor of the combined endpoint as well as total mortality alone in AF patients (HR 2.10, CI 1.28–3.45, *p* = 0.003 and HR 2.18, CI 1.32–3.60, *p* = 0.002, respectively). AF patients with BivP> 98% did not demonstrate significant differences in prognosis compared to patients with no AF when either the combined endpoint or total mortality was used as outcome (HR 1.52, CI 0.89–2.60, *p* = 0.12 and HR 1.58 CI 0.92–2.71, *p* = 0.100, respectively).

## Discussion

In our cohort of HF patients with CRT, mostly consisting of CRT-P recipients, a history of AF before CRT implantation was associated with an unfavorable outcome, thus supporting earlier observations [3, 4, 16, 17]. However, when adjusted for clinical relevant covariates, AF history was only borderline significant as a predictor of the combined endpoint and total mortality suggesting that AF is an important marker of a more advanced disease but may not influence the outcome by itself. On the other hand, adequate BivP appeared to diminish deleterious impact of AF on prognosis since prognostic impact of AF-history was confined to those who did not have BivP extent exceeding 98% while AF-patients with BivP> 98% had prognosis similar to CRT-treated HF patients without AF.

### Prevalence of pre-procedural atrial fibrillation

We report higher-than-expected prevalence of pre-procedural AF (54%), which is considerably higher than in earlier observations from CRT studies [3, 4, 17, 18]. However, our cohort was very symptomatic as 91% of all patients were in NYHA Class III or IV. Compared to the previous studies that observed up to 50% AF prevalence in patients with an advanced heart failure [4], we believe that AF prevalence reported in our CRT cohort is in line with what one would expect in a symptomatic cohort of heart failure patients thus indicating less biased selection for CRT implantation. Furthermore, it is reasonable to believe that data collection from different sources has contributed to a higher AF detection rate translated in a higher AF prevalence estimates. Besides, the SNPR has been found to be a reliable source of information regarding AF [13] that may also account for some of the study’s unusually high AF-prevalence. Eight per cent of our patients had earlier undocumented AF revealed from the digital ECG archive review, which was not reflected in either the SNPR or medical records, thus indicating that the prevalence of pre-procedural non-permanent AF history may be underestimated in CRT-cohorts.

### Incidence and prognostic impact of device-detected atrial fibrillation during the first year of follow-up

The incidence of device-detected AF has previously been found to be high in patients with CRT and no pre-procedural AF [11] and our study is in agreement with previous findings as the one-year incidence of device-detected AF in patients with no AF before CRT implantation was as high as 22%. In patients with no pre-procedural AF history, device-detected AF during first year of follow-up was not associated with poor outcome in our material. However, it is possible that our study is underpowered to reveal a true association of device-detected AF and unfavorable outcome, since previous studies have shown conflicting results regarding the prognostic importance of device-detected AF[11, 12]. As AF may cause loss of BivP and non-response to treatment in CRT-patients [4, 9], it may be particularly important to diagnose in this group of patients. The high degree of uncertainty regarding the often-found device-detected AF underlines the need of more studies.

### Prognostic impact of achieved biventricular pacing during the first year of follow-up

As AF history by one year after CRT implantation and BivP≤98% during first year of follow-up independently predicted unfavorable outcome, adequate BivP certainly seems to be a major determinant of CRT-outcome, corresponding to earlier findings [8, 9]. Also, AF-subjects with BivP> 98% did not have worse outcome compared to patients with no AF, further strengthening the notion that achieved BivP is an important predictor of CRT-outcome in patients with AF.

It is not surprising that the achieved BivP during follow-up is lower in our cohort in patients with a higher burden of AF as it is known that it can be troublesome to achieve adequate BivP in patients with AF and our results are thus in agreement with previous findings [7, 9].

AF-rhythm with fast ventricular rates may interfere with adequate BivP delivery [4]. However, we did not observe significant differences in baseline heart rate between AF patients with BivP > 98% compared to those with BivP ≤98% at one-year follow-up. As atrial tachyarrhythmia can be an important cause of BivP loss [7], and that our patients with AF had less BivP by the first year of follow-up, suggests that arrhythmia burden during follow-up is an important determinant of the ability to achieve adequate BivP. When comparing patients with non-permanent and permanent AF, patients with permanent AF had a higher likelihood of a BivP ≤98% (57% vs. 39%, *p*-value 0,04). The median AF burden in the non-permanent AF group was surprisingly low (IQR 0–15%) during the first year of CRT treatment in our group of patients. The low AF burden may partly be explained by the fact that some patient’s pre procedural AF diagnosis was based only on manual ECG review. One may also speculate if this finding of low AF burden is due to reduced atrial pressure which in turn may lead to a lower AF burden. It has previously been shown that the AF burden among patient with non-permanent AF can be reduced during the first three months of CRT treatment [19].However, as no other differences in baseline characteristics that may influence outcome were observed between groups above or below 98% BivP, we believe that our results strongly support the notion that a high percentage of BivP is a major determinant of CRT-outcome.

### Limitations

We did not study intra-cardiac electrograms, there was no standardized way to keep the physicians’ record of device-stored data, and it is possible that some AHREs do not in fact reflect true episodes of AF. However, our estimates of device detected AF are in line with previous observations and since AHREs and AMS algorithms have been found to be trustworthy surrogate markers for AF in CRT-pacemakers [20], we believe that a vast majority of episodes indeed reflects AF. Another obvious limitation to the study is the fact that only 254 of all 379 patients had enough available data to be included in the analyses regarding the prognostic impact of BivP. However, we do not believe that this caused any significant selection-bias. Regarding the study population the decisions were based on the guidelines that were in force during the period when patients were treated but it is probable that patient-physician preferences might have influenced those decisions causing a non-homogenous group of patients as reflected in Table 1.

## Conclusions

In patients with congestive heart failure treated with CRT, AF is a common comorbidity, associated with poor outcome, and its prevalence is likely to be underestimated at the time of device implantation. AF history is associated with higher mortality, but does not independently predict mortality or disease progression to heart transplantation, signaling that it is most likely only a marker of a more advanced disease and/or other comorbid conditions. Insufficient extent of BivP≤98% during the first year of follow-up is an independent predictor or poor outcome thus further supporting the use of 98% threshold of BivP, which should be attained in order to maximize the benefits of CRT.

## Data Availability

The datasets used and/or analyzed during the current study are available from the corresponding author on reasonable request.

## Abbreviations

AF: Atrial fibrillation
AHRE: Atrial high rate episodes
AMS: Atrial mode switching
AV: Atrioventricular
BivP: Biventricular pacing
CI: Confidence interval
CRT: Cardiac resynchronization therapy
CRT-D: Cardiac resynchronization therapy with defibrillator
CRT-P: Cardiac resynchronization therapy without defibrillator
ECG: Electrocardiogram
HR: Hazard ratio
LBBB: Left bundle branch block
LVEF: Left ventricular ejection fraction
NYHA: New York heart association
SNPR: Swedish national patient register
